# A combined linkage, microarray and exome analysis suggests *MAP3K11* as a candidate gene for left ventricular hypertrophy

**DOI:** 10.1186/s12920-018-0339-9

**Published:** 2018-03-05

**Authors:** Claudia Tamar Silva, Irina V. Zorkoltseva, Maartje N. Niemeijer, Marten E. van den Berg, Najaf Amin, Ayşe Demirkan, Elisa van Leeuwen, Adriana I. Iglesias, Laura B. Piñeros-Hernández, Carlos M. Restrepo, Jan A. Kors, Anatoly V. Kirichenko, Rob Willemsen, Ben A. Oostra, Bruno H. Stricker, André G. Uitterlinden, Tatiana I. Axenovich, Cornelia M. van Duijn, Aaron Isaacs

**Affiliations:** 1 Genetic Epidemiology Unit, Department of Epidemiology, Erasmus University Medical Center, Rotterdam, the Netherlands; 2 Center for Research in Genetics and Genomics (CIGGUR), Institute of Translational Medicine (IMT), GENIUROS Research group, School of Medicine and Health Science, Universidad del Rosario, Bogotá, Colombia; 3 Doctoral Program in Biomedical Sciences, Universidad del Rosario, Bogotá, Colombia; 4 Institute of Cytology and Genetics SD RAS, Novosibirsk, Russia; 5 Department of Epidemiology, Erasmus University Medical Center, Rotterdam, the Netherlands; 6 Department of Medical Informatics, Erasmus University Medical Center, Rotterdam, the Netherlands; 7 Department of Clinical Genetics, Erasmus University Medical Center, Rotterdam, the Netherlands; 8 Center for Medical Systems Biology, Leiden, the Netherlands; 9 Department of Internal Medicine, Erasmus University Medical Center, Rotterdam, the Netherlands; 10 Inspectorate of Health care, The Hague, the Netherlands; 11 CARIM School for Cardiovascular Diseases, Maastricht Centre for Systems Biology (MaCSBio), and Department of Biochemistry, Maastricht University, Maastricht, the Netherlands

## Abstract

**Background:**

Electrocardiographic measures of left ventricular hypertrophy (LVH) are used as predictors of cardiovascular risk. We combined linkage and association analyses to discover novel rare genetic variants involved in three such measures and two principal components derived from them.

**Methods:**

The study was conducted among participants from the Erasmus Rucphen Family Study (ERF), a Dutch family-based sample from the southwestern Netherlands. Variance components linkage analyses were performed using Merlin. Regions of interest (LOD > 1.9) were fine-mapped using microarray and exome sequence data.

**Results:**

We observed one significant LOD score for the second principal component on chromosome 15 (LOD score = 3.01) and 12 suggestive LOD scores. Several loci contained variants identified in GWAS for these traits; however, these did not explain the linkage peaks, nor did other common variants. Exome sequence data identified two associated variants after multiple testing corrections were applied.

**Conclusions:**

We did not find common SNPs explaining these linkage signals. Exome sequencing uncovered a relatively rare variant in *MAPK3K11* on chromosome 11 (MAF = 0.01) that helped account for the suggestive linkage peak observed for the first principal component. Conditional analysis revealed a drop in LOD from 2.01 to 0.88 for *MAP3K11,* suggesting that this variant may partially explain the linkage signal at this chromosomal location. MAP3K11 is related to the JNK pathway and is a pro-apoptotic kinase that plays an important role in the induction of cardiomyocyte apoptosis in various pathologies, including LVH.

**Electronic supplementary material:**

The online version of this article (10.1186/s12920-018-0339-9) contains supplementary material, which is available to authorized users.

## Background

Left ventricular hypertrophy (LVH) is a predictor of increased cardiovascular morbidity and mortality [1]. Those with LVH have a 2-fold increased risk of adverse events, particularly ischemic heart disease and chronic heart failure [2, 3]. Increased left ventricular mass maintains cardiac pump performance in response to cardiovascular insults, such as coronary heart disease [3, 4]. Risk factors for LVH are elevated systolic blood pressure, obesity, hypertension, insulin resistance, valvular heart disease and advanced age, among others [2, 5, 6]. LVH proxy measurements can be assessed through noninvasive methods, such as echocardiography and magnetic resonance imaging, however, electrocardiographic measurements are the most used worldwide [7]. LVH proxy measurements include calculations of the Sokolow-Lyon index (SL), the Cornell voltage product (CV) and the 12-lead sum QRS product (12LS). Several studies have demonstrated that genetic factors influence electrocardiographic and echocardiographic measures of LVH [2, 4, 5, 8, 9]. We recently demonstrated that these measures contain a substantial heritable component (SL = 0.46, 12LS = 0.49 and CV = 0.34) [10].

Genome-wide linkage analyses, candidate gene association studies, genome-wide association studies (GWAS) and gene mapping have been conducted to identify genes influencing LVH. In the first GWAS of these traits, two loci, *PTGES3* and *NMB,* reached genome-wide significance. *IGF1R* and *SCN5A* were identified and replicated without reaching genome-wide significance [5]. Recently, an expanded GWAS detected a number of novel loci influencing CV, SL, and 12LS [11]. Among these were 32 loci containing genes with known cardiac function, coding for cardiac sarcomere components or related to cardiac myocyte function. Evidence for linkage of echocardiographic LV mass to chromosome 5 (LOD score = 1.6) and electrocardiographic LV mass to chromosome 7 (LOD score = 1.67) [8] and chromosome 12 (LOD score = 2.19 and 3.11) [8, 12] were reported in linkage studies, with the strongest evidence for chromosome 12 [3]. As is the case for other complex outcomes, most candidate genes studies have not been replicated and do not reach genome-wide significance [3].

Exome sequencing has been successfully used for Mendelian disorders [13]. More recently, this technology has been extended to the analysis of non-Mendelian diseases and complex traits, as rare variants with large effects can contribute to the heritability of common traits. The aim of this study was to discover rare variants by linkage analysis in a large family-based study, the Erasmus Rucphen Family (ERF) study. Linked regions were fine-mapped in detail using microarray data and exome sequencing.

## Methods

### Study population

The ERF study is a family-based study including over 3000 participants descendant from 22 couples that lived in the Rucphen region in the southwest Netherlands in the nineteenth century [14]. All descendants of those couples were invited to visit the clinical research center in the region where they were examined in person [15]. Interviews at the time of blood sampling were performed by medical practitioners and included questions on current medication use and medical history [16]. Additionally, participants were asked to bring their current medications with them to the study center; these were cross-referenced with general practitioner and pharmacy records. Height and weight were measured with the participant in light underclothing and body mass index (kg/m^2^) was computed. Blood pressure was measured twice on the right arm in a sitting position after at least 5 min rest, using an automated device (OMRON 711, Omron Healthcare, Bannockburn, IL, USA). The average of the two measures was used for analysis. Hypertension status was identified through the use of antihypertensive medication and/or through the assessment of blood pressure measurements according to the guidelines of the World Health Organization [17]. The Medical Ethics Committee of the Erasmus University Medical Center approved the ERF study protocol and all participants, or their legal representatives, provided written informed consent.

### ECG interpretation and measurements

Examinations included 12-lead ECG measurements. A 10 s 12-lead ECG (on average, 8 to 10 beats) was recorded with an ACTA-ECG electrocardiograph (Esaote, Florence, Italy) with a sampling frequency of 500 Hz. Digital measurements of the ECG parameters were made using the Modular ECG Analysis System (MEANS) [18, 19]. Briefly, MEANS operates on multiple simultaneously recorded leads, which are transformed to a detection function that brings out the QRS complex and the other parts of the signal. MEANS determines common onsets and offsets for all 12 leads together on one representative averaged beat, with the use of template matching techniques. The measurement and diagnostic performance of MEANS has been extensively evaluated, both by the developers and by others [19–22]. The MEANS criteria for MI are mainly based on pathological Q waves, QR ratio, and R-wave progression [20]. A cardiologist, specialized in ECG methodology, ascertained the final diagnosis of MI.

MEANS was used to measure QRS complex duration and the three LVH proxies. Sokolow-Lyon was defined as the sum of the S wave in V1 plus the R wave in V5 or V6, Cornell as the sum of R in aVL and S in V3, and 12-lead as the sum of R to S in all 12 leads; these three voltages were then multiplied by QRS duration to obtain voltage-duration products as an approximation of the area under the QRS complex [21–23]. Principal component (PC) analysis was applied to the three original measurements (SL, 12LS and CV) to capture the correlation structure between traits. Two PCs, PC1 and PC2, captured more than 94% of the total variance and were also assessed as phenotypes in these analyses. All traits were adjusted for sex, age, BMI and height and the residuals were rank transformed prior to analysis.

### Genotyping and statistical analysis of the linkage study

Illumina’s HumanHap6k Genotyping BeadChip (*6 K Illumina Linkage IV Panels®*) was used for genotyping for the linkage analyses. All genotyping procedures were performed according to the manufacturer’s protocols. Only markers with a minor allele frequency (MAF) > 0.05 were selected for further analysis. Genotyping errors leading to Mendelian inconsistencies were detected using PedCheck [24]. Unlikely double recombination events were detected using MERLIN [25]. All detected errors were eliminated from the data. A total of 5250 autosomal SNPs with a call rate greater than 95% were utilized for the linkage analyses. Among the 2385 individuals who were phenotyped for LVH measures, 1860 people also had genotype data and were included in the linkage study. Variance component multipoint linkage was performed using the --vc option in MERLIN v.1.0.1 [25, 26]. This program calculates exact IBD sharing probabilities using the Lander-Green algorithm, requiring restrictions on pedigree size. Because of this, the single ERF pedigree with multiple loops was split into non-overlapping fragments of no more than 18 bits with the help of the PedSTR program [27].

Regions of interest with LOD > 1.9 were selected for further analysis. Borders of the linkage areas were defined as LOD score minus 2 support intervals (LOD-2 SI) around the linkage peaks. Genes within the LOD-2 SI were annotated using SCAN (SNP and CNV Annotation Database).

### Genotyping and statistical analysis of the association study

Of 2385 phenotyped people, dense genotypes were available for 2128 subjects, typed on 3 different genotyping platforms (Illumina 318 K, Illumina 370 K and Affymetrix 250 K), which were merged first (median number of quality controlled SNPs per individual = 325,500) and then ~ 2.54 million SNPs were imputed using MACH (v1.0.16) [28, 29], with the HapMap build 36 (release 22) CEU population as reference. Within each genotyping batch, only SNPs with a call rate > 98%, MAF > 1% and Hardy-Weinberg Equilibrium *P*-value > 10^− 6^ were used for imputations. To account for relatedness, a genomic kinship matrix was computed in GenABEL [30]. This matrix was then incorporated into linear mixed-effects regression models, as implemented in ProbABEL [31], which were used to assess the association of variants in the LOD-2 SI with the LVH phenotypes. *P*-values were adjusted with the FDR-based q-value technique [32].

### Exome sequencing

The exomes of 1336 individual from the ERF population were sequenced “in-house” at the Center for Biomics of the Department of Cell Biology of the Erasmus MC, the Netherlands, using the Agilent version V4 capture kit on an Illumina HiSeq 2000 sequencer using the TruSeq Version 3 protocol. Mean depth base was 74.23× (median = 57×) and mean depth region was 65.26× (median = 52.87×). The sequence reads were aligned to the human genome build 19 (hg19) using BWA and the NARWHAL pipeline [33, 34]. The aligned reads were processed further using the IndelRealigner, MarkDuplicates and TableRecalibration tools from the Genome Analysis Toolkit (GATK) and Picard (http://broadinstitute.github.io/picard/) to remove systematic biases and to recalibrate the PHRED quality scores in the alignments. Genetic variants were called using the Unified Genotyper tool of the GATK. About 1.4 million Single Nucleotide Variants (SNVs) were called and, after removing the low quality variants (QUAL < 150), we retrieved 577,703 SNVs in 1309 individuals. ECG and covariate data were available for 1072 of these samples. Further, for comparison and to predict the functionality of the variants, annotations were also performed using the dbNSFP (database of human non-synonymous SNPs and their functional predictions, http://varianttools.sourceforge.net/Annotation/DbNSFP) and Seattle (http://snp.gs.washington.edu/SeattleSeqAnnotation138/) databases. These databases gave functional prediction results from four different programs, PolyPhen-2, SIFT, MutationTaster and LRT, apart from gene and variant annotations.

We employed a Bonferroni correction for the number of deleterious mutations selected for each trait to correct for multiple comparisons in the exome data: 101 for SL (*P*-value = 4.9 × 10^− 4^), 98 for CV (*P*-value = 5.1 × 10^− 4^) and 60 for 12 LS (*P*-value = 8.3 × 10^− 4^). For the PCs, the numbers were 141 for PC1 (*P*-value = 3.5 × 10^− 4^) and 71 for PC2 (*P*-value = 7.0 × 10^− 4^).

### Replication

Four SNPs (rs139580877, rs138968470, rs35996030 and rs142551296) were selected for replication in the Rotterdam Study (RS). The Rotterdam Study is a prospective cohort study ongoing since 1990 in the city of Rotterdam in the Netherlands [35].

Exomes from 1764 individuals from the RS population were sequenced at an average depth of 20× using the Nimblegen SeqCap EZ V2 capture kit on an Illumina HiSeq 2000 sequencer and the TrueSeq Version 3 protocol. The sequence reads were aligned to hg19 using BWA. Subsequently, the aligned reads were processed further using Picard, SAMtools and GATK. Genetic variants were called using the Unified Genotyper Tool from GATK. Samples with low concordance to genotyping array (< 95%), low transition/transversion ratio (< 2.3), high heterozygote to homozygote ratio (> 2.0) and low call rate (< 80%) were removed from the data. SNVs with a low call rate (< 90%) and out of HWE (*P*-value < 10^− 6^) were also removed from the data. The final dataset consisted of 635,814 SNVs in 1450 individuals with complete phenotype and covariate data.

One SNP, rs139580877, was not available in the Rotterdam Study exome data. This variant was imputed using the GIANT 1000 Genomes Phase I Version 3 All reference panel, as previously described [36]. In brief, after filtering SNPs genotyped with the Illumina v3 Infinium II HumanHap550 microarray for deviations from Hardy-Weinberg proportions (*P* < 1 × 10^− 6^_)_, call rate (< 98%), MAF (< 0.01), and Mendelian errors (> 100), MACH was used to perform the imputations.

## Results

**Fig. 1:**
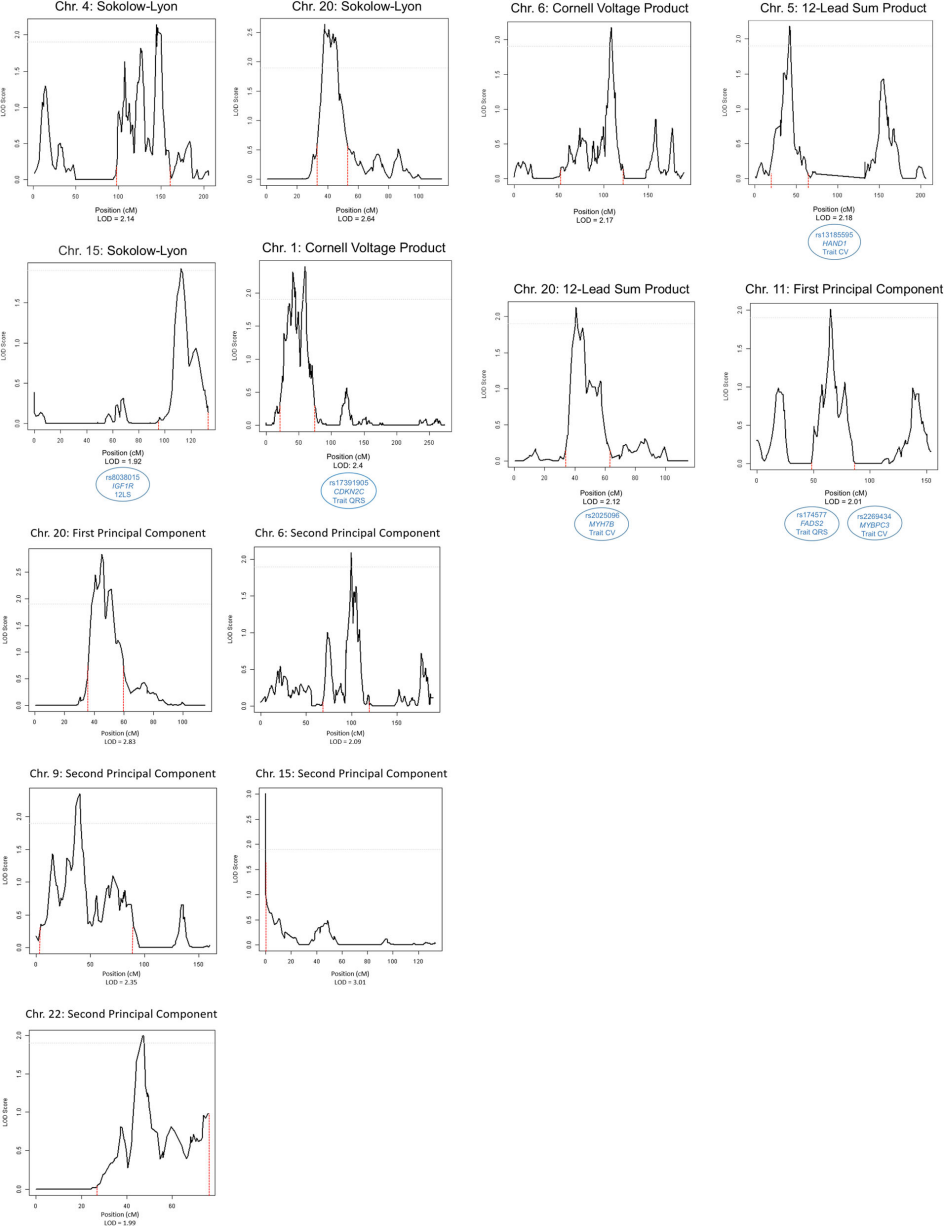
Linkage peaks for the LVH proxy measures. Plots depicting the linked regions by trait and chromosome. The grey dashed horizontal line indicates the threshold for suggestive linkage. The red dashed vertical lines show the borders of the LOD score minus 2 support intervals (LOD-2 SI). The blue circles contain SNPs identified in previous GWAS for these traits in the LOD-2 SI

Table 1 shows characteristics of the participants in the LVH linkage, microarray, and exome sequence analyses. The proportion of LVH cases for each proxy measure was determined using published cut-off values [37, 38]. There were no significant differences between these overlapping groups. Table 2 shows the correlation between the traits (r = 0.76 in the adjusted model for SL and 12LS, 0.17 between SL and CV, and 0.48 for CV and 12LS). Table 3 shows the loadings of the three LVH proxies (SL, CV, 12LS) to the two PCs that were constructed. PC1 predominantly captured SL and 12LS, while PC2 correlated strongly with CV and moderately with SL. Table 4 shows the linkage results for the LVH proxy measures, which yielded a total of seven regions with suggestive LOD scores (LOD > 1.9). SL was linked to three regions, with the highest LOD score for chromosome 20 (LOD = 2.64) and two additional regions on chromosomes 4 (LOD = 2.14) and 15 (LOD = 1.92). Suggestive LOD scores for CV were seen on chromosomes 1 (LOD = 2.4) and 6 (LOD = 2.17). There was suggestive linkage of 12LS to chromosomes 5 (LOD = 2.18) and 20 (LOD = 2.12). Linkage results for the principal component analysis of the LVH measures showed one significant LOD score for PC2 on 15q11.2 (LOD = 3.01). This region was also linked to SL (LOD = 1.92). Two regions were suggestively linked to PC1: 11q13.4 (LOD = 2.01) and 20p12.1 (LOD = 2.83), which was also linked to SL and 12LS. For PC2, there were three suggestive linkage results, for chromosomes 6 (LOD = 2.09), 9 (LOD = 2.35) and 22 (LOD = 1.99). The chromosome 6 region was also linked to CV. Plots showing the linked regions by chromosome are provided in Fig. 1. Table 5 shows the top common variant microarray-based association signals under the LVH trait linkage peaks, including *P*-values and MAF for each SNP. None achieved statistical significance after correction for multiple comparisons.

**Table 1:** Descriptive statistics of the Erasmus Rucphen Family (ERF) study population

	Microarray			Linkage			Exon sequence		
	*n* = 2128			*n* = 1860			*n* = 1072		
	Mean (S.D.)	Minimum	Maximum	Mean (S.D.)	Minimum	Maximum	Mean (S.D.)	Minimum	Maximum
Males	899 (42%)			775 (42%)			408 (38%)		
Age (y)	47.0 (13.82)	16.6	85.3	46.5 (13.79)	16.6	85.3	46.51 (13.7)	18.7	81.0
BMI (kg/m^2^)	26.7 (4.57)	15.5	61.8	26.7 (4.58)	15.5	61.8	26.4 (4.3)	15.5	61.8
Height (cm)	167.6 (9.31)	139.3	196.5	167.4 (9.19)	143.6	196.5	166.7 (9.0)	143.6	196.5
Weight (kg)	75.1 (15.16)	41.9	161.0	74.9 (15.5)	41.9	161	73.6 (14.3)	42.1	161.0
SBP (mm Hg)	138.4 (19.5)	85.5	222.0	137.7 (19.1)	85.5	217.0	137.0 (18.7)	85.5	216.0
DBP (mm Hg)	79.9 (9.8)	53.5	124.0	79.7 (9.7)	54.5	120.0	79.1 (9.6)	53.5	120.0
Hypertension	913 (43%)			766 (42%)			549 (51%)		
SL	2344 (690.6)	884.0	5288.0	2341 (690.6)	884	52.9	2319 (659.0)	967	5288.0
CV	1173.5 (505.1)	93.1	4126.1	1170.0 (497.3)	93.1	3952.8	1151.6 (659.0)	155.8	3853.0
12LS	13,862 (3812.3)	4993	39,250	13,805 (3767.8)	49.9	39.2	13,610.0 (3628.7)	5485.0	36,364
LVH (SL)	138 (6.5%)			120 (6.4%)			66 (6.2%)		
LVH (CV)	41 (1.9%)			32 (1.7%)			20 (1.9%)		
LVH (12LS)	176 (8.3%)			147 (7.9%)			76 (7.1%)		

Values presented are mean (standard deviation) or n (%)

*BMI* body mass index, *SBP* systolic blood pressure, *DBP* diastolic blood pressure, *SL* Sokolow-Lyon index, *CV* Cornell product, *12LS* 12-lead sum product

**Table 2:** Pearson’s correlations between LVH proxy measures

	Unadjusted	Adjusted
SL – 12LS	0.80	0.76
SL – CV	0.29	0.17
CV – 12LS	0.56	0.48

Adjusted model included age, sex, body-mass index, and height

*SL* Sokolow-Lyon, *CV* Cornell Voltage product, *12LS* twelve-lead sum product, *PC1* first principal component, *PC2* second principal component

**Table 3:** PC loadings for LVH proxies

	Principal Component	
	PC1	PC2
SL	0.84	−0.48
CV	0.61	0.78
12LS	0.95	−0.08

Adjusted model included age, sex, body-mass index, and height

*SL* Sokolow-Lyon, *CV* Cornell Voltage product, *12LS* twelve-lead sum product, *PC1* first principal component, *PC2* second principal component

**Table 4:** Results of the linkage analyses

Trait	N	Chr.	SNP	LOD-2 SI Lower	LOD-2 SI Upper	Position (cM)	LOD_MAX_
SL	1860	4	rs1032328	90,166,159	157,272,456	144.46	2.14
SL	1860	15	rs290370	88,026,435	102,212,431	112.3	1.92
SL	1860	20	rs204115	11,094,951	23,352,685	38.11	2.64
CV	1860	1	rs6619	12,296,232	53,396,842	59.63	2.40
CV	1860	6	rs2040431	72,253,060	117,799,468	108.31	2.17
12LS	1860	5	rs1442470	7,205,420	25,399,905	42.3	2.18
12LS	1860	20	rs466243	11,017,796	23,352,685	40.7	2.12
PC1	1860	11	rs1530354	33,896,047	78,743,080	65.21	2.01
PC1	1860	20	rs2077147	11,990,037	38,247,165	45.09	2.83
PC2	1860	6	rs1391503	87,511,828	105,402,837	99.69	2.09
PC2	1860	9	rs748530	8,378,662	25,788,723	40.22	2.35
PC2	1860	15	rs1562203	–	23,707,591	0	3.01
PC2	1860	22	rs138383	35,687,558	44,707,606	46.89	1.99

Model adjusted for age, sex, body-mass index, and height

*SL* Sokolow-Lyon, *CV* Cornell Voltage product, *12LS* twelve-lead sum product, *PC1* first principal component, *PC2* second principal component, *N* sample size, *Chr.* chromosome, *LOD-2 SI Lower* position of lower boundary of support interval in base pairs, *LOD-2 SI Upper* position of upper boundary of support interval in base pairs, *LOD*_*MAX*_ LOD score at SNP

**Table 5:** Top association signals from the microarray data under the LVH trait linkage peaks

Outcome	Region	SNP	MAF	Gene	*P*-value	Q-value
SL	4q26	rs6839953	0.27	*TRAM1L1*	1.34 × 10^−4^	0.47
SL	15q26.2	rs11074275	0.48	*MCTP2*	4.27 × 10^−4^	0.79
SL	20p12.1	rs721243	0.19	*ISM1*	7.37 × 10^−5^	0.15
CV	1p35.1	rs16835131	0.06	*SYNC*	1.35 × 10^−5^	0.35
CV	6q15	rs10944412	0.27	*RNGTT*	4.60 × 10^−5^	0.93
12LS	5p15.2	rs2589661	0.10	*ROPN1L*	1.26 × 10^− 4^	0.46
12LS	20p11.23	rs6106235	0.18	*C20orf26*	1.69 × 10^−5^	0.09
PC1	11q12.2	rs1790325	0.04	*FADS1*	2.85 × 10^−5^	0.08
PC1	20p12.1	rs13036282	0.005	*SPTLC3*	2.30 × 10^−4^	0.63
PC2	6q16.3	rs1475922	0.06	*GRIK2*	1.64 × 10^−4^	0.94
PC2	9p24.1	rs10975939	0.003	*KDM4C*	4.67 × 10^−4^	1.00
PC2	15q11.2	rs8043191	0.03	*CYFIP1*	5.95 × 10^−3^	0.52
PC2	22q13.33	rs2688089	0.45	*C22orf34*	7.02 × 10^−5^	0.56

*SL* Sokolow-Lyon, *CV* Cornell Voltage product, *12LS* twelve-lead sum product, *PC1* first principal component, *PC2* second principal component, *MAF* minor allele frequency

### Variants in the coding sequence

**Fig. 2:**
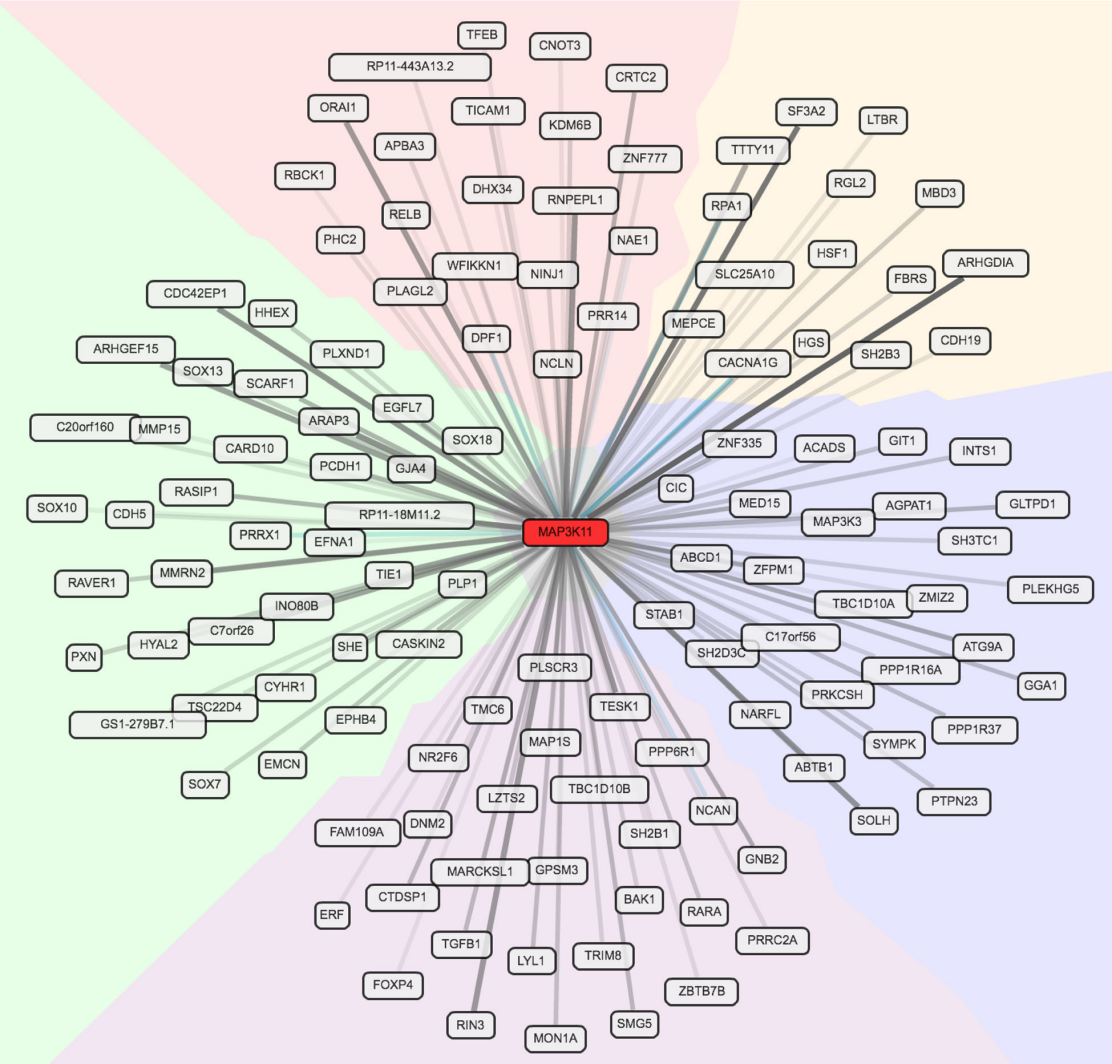
MAP3K11 gene network interactions. Looking for interactions for MAP3K11, we searched Gene Network (http://genenetwork.nl/gene/ENSG00000173327). One hundred twenty-nine gene-gene interactions are shown

The results of the search for less frequent exonic variants are summarized in Additional file 1: Table S1. We focused on relatively rare (frequency < 5%) missense variants predicted to be deleterious by at least two of the prediction algorithms used and non-sense variants. This selection yielded 471 variants in 356 genes in the 13 linkage intervals (LOD-2 SI), which we analysed with respect to the LVH proxy measures and PCs. Additional file 1: Table S2 shows the results with a nominal *P-*value ≤0.05 after regressing out the effects of age, BMI, height and sex. This effort uncovered an A > G variation (rs139580877) in the *SPEF2* gene on 5p13.2, which was significantly associated with 12LS when adjusted for multiple testing (*P*-value = 4.2 × 10^− 4^). This variant, with 108 carriers in ERF, is predicted to be probably damaging by PolyPhen-2 with a score of 0.972 and as deleterious by SIFT with a score ranging between 0.02 and 0.03. It is a missense variant, among more than 2000 described for this gene. In the principal components analysis, rs138968470, on 11q13.1 in the *MAP3K11* gene, was associated with PC1 adjusted for multiple testing (*P*-value = 3.5 × 10^− 4^). SKAT-O and burden tests provided some supporting evidence for the association of this gene with LVH proxy measures (Additional file 1: Table S3). Additionally, at the SL chromosome 4 locus, we identified a C > G variation (rs142551296) in *PRSS12* that approached significance (*P*-value = 8.4 × 10^− 4^). A second, more common intragenic variant inside *PRSS12* was nominally associated (rs35996030; *P*-value = 0.04). We re-ran the linkage analyses conditioning on these variants to see if they explained the observed linkage signals. For PC1, the LOD score in the 11q13.4 linkage region dropped in the conditional analysis (from 2.01 to 0.88), suggesting that the associated variant (rs138968470), or neighbouring variants in linkage disequilibrium (LD), explained the linkage signal. This variant also showed evidence of association with the two traits (12LS and SL) underlying PC1 (*P*-value = 3.0 × 10^− 4^ and *P*-value = 1.2 × 10^− 3^, respectively). Using Gene Network (http://genenetwork.nl/gene/ENSG00000173327), to perform in-depth analyses of the expression of *MAP3K11*, demonstrated that its expression is strongly linked to rho signalling (*ARGHGEF15, ARHGDIA*) (Fig. 2).

Five of the linkage peaks contained loci recently identified in GWAS studies [5, 11]. To determine if the linkage signals were a result of those common variants, linkage was performed a second time, conditioned on the GWAS index SNPs. These analyses demonstrated that the observed peaks were not explained by the GWAS SNPs, although the estimates fluctuated somewhat, likely as a result of smaller sample sizes (Additional file 1: Table S4).

### Replication

Summary statistics for the Rotterdam Study sample are provided in Additional file 1: Table S5. The variant rs139580877 was imputed, using the 1000 Genomes reference panel; the imputation quality score (MACH RSQ) for this variant was 0.65, with a minor allele frequency of 0.008. The effect estimate for 12LS was essentially zero, and therefore, did not replicate the ERF findings (Additional file 1: Table S6). The other variants of interest, rs35996030, rs138968470 and rs142551296, were directly genotyped in a subset of the Rotterdam Study cohort (*n* = 1450). There was no evidence of association for any of these variants in the Rotterdam Study.

## Discussion

We performed a linkage study on LVH proxy measurements, and PCs, and identified one significant locus (15q11.2) and 10 suggestive regions (1p34, 4q31, 5p14, 6q15, 6q21, 9p21, 11q13.4, 15q25, 20p12, 22q13). Exome variant analysis in these regions uncovered a missense coding variation in *MAP3K11* on 11q134 for PC1; the *MAP3K11* variant substantially decreased the LOD score for this peak. The 24 carriers of this missense mutation clustered into five pedigrees in the ERF population (Additional file 1: Figure S2).

Genetic variants discovered by GWAS, based on individual single-nucleotide polymorphisms (SNPs), explain only a small proportion of the heritability of complex traits [10, 39, 40]; we found variants with larger effect sizes compared to the ones found with GWAS. Our analysis of rare coding variants in these linkage regions revealed a variant, rs138968470 on 11q13.1 in the *MAP3K11* gene, associated with PC1. Conditional linkage analysis, including the *MAP3K11* variant, reduced the LOD score (from 2.01 to 0.88), suggesting that this variant largely explained the linkage signal at this chromosomal location. The SNP is located in the first exon of a gene encoding a protein that belongs to the serine/threonine kinase family of mitogen-activated protein kinases. MAP3K11 (also known as Mixed Lineage Kinase 3 (MLK3)) [34], works as a positive regulator of the c-Jun N-terminal kinase (JNK) signalling pathway [41]. MAP3K11 has a CDC42 and Rac interacting proteins binding domain (CRIB); autophosphorylation of MAP3K11 and the induction of JNK is mediated through this CRIB domain bound to Cdc42/Rac/GTP [42]. JNK, an important member of the mitogen-activated protein kinase family (MAPK), is a pro-apoptotic kinase that plays an important role in the induction of cardiomyocyte apoptosis in various pathologies [43]. Apoptosis increases with LVH, a critical mechanism that mediates the transition from compensated hypertrophy to heart failure [44]. In this way, a damaging mutation in *MAP3K11* may be related to regulation of JNK and the subsequent JNK controlled pathway.

The other significant missense variant was rs139580877, located on 5p14. This variant is in exon 9 of the gene that encodes the sperm flagellar protein (S*PEF2)*, which has been postulated to play an important role in spermatogenesis and flagellar assembly [45]. This SNP was not found to be responsible for the linkage signal in the region, despite its strong association. The association with this relatively common variant (MAF = 0.015) could not be confirmed in the Rotterdam Study. One additional finding was studied further: a C/G variant (rs142551296) in the *PRSS12* gene, underlying the SL locus on chromosome 4, which approached significance (*P*-value = 8.4 × 10^− 4^), but did not replicate in the Rotterdam Study. Absence of replication could be related to imputation quality for rs139580877 and the low number of carriers for the other SNPs (Additional file 1: Table S4).

A number of the linkage peaks contained SNPs identified in a large GWAS of these traits. Linkage analysis, conditioned on the index SNPs from the GWAS, did not significantly alter the linkage results. This suggests that the linkage peaks were not driven by the common variants identified in the GWAS.

No explanatory variants were found for most of the loci (suggestively) linked to LVH, for which there are a number of potential explanations. Linkage peaks are not precise in highlighting the location of the causal variant; even the region of interest cannot be easily pinpointed. Additionally, we did not take into account alternative mechanisms, such as structural and copy number variations (CNVs) or repeats in the linkage regions. Lastly, causal rare variants may be located outside the coding sequence, which we did not include in our sequencing analyses.

## Conclusions

In conclusion, 13 loci were identified for ECG LVH proxy measures and PCs using linkage analysis in a large pedigree; these were subsequently fine-mapped with microarray and exome sequence data. Common variation from the microarrays did not explain these peaks. The exome data, though, suggested the involvement of *MAP3K11* (11q13) in LVH through the regulation of JNK. However, we cannot exclude the presence of other variants that are in linkage disequilibrium with the *MAP3K11* variant (rs138968470) that might explain the observed association.

Further analysis will need to be performed to demonstrate the involvement of this protein in LVH. A number of other suggestively linked peaks were determined. We could not explain these with microarray or exonic sequence variants at present, asking for more extensive follow-up outside the coding regions.

## Additional file

Additional file 1: **Table S1.** Coding variants under the linkage peaks for LVH proxy measurements. **Table S2.** Selected damaging variants in the coding regions contained in the linkage regions. **Table S3.** SKAT and burden tests for genes of interest. **Table S4.** Results of linkage analyses before (LOD1) and after (LOD2) regression on GWAS SNPs under the linkage peaks. **Table S5.** Descriptive statistics of the Rotterdam study population. **Table S6.** Replications results in the Rotterdam Study. **Figure S1.** Venn diagram showing the overlap between the different ERF genotyping experiments. **Figure S2.** Pedigrees segregating rs138968470. (DOCX 119 kb)

## Abbreviations

12LS: 12-lead sum QRS product
BMI: Body mass index
CRIB: CDC42 and Rac interacting proteins binding domain
CV: Cornell voltage product
ECG: Electrocardiogram
ERF: Erasmus Rucphen Family Study
GATK: Genome Analysis Toolkit
GWAS: Genome Wide Association Studies
HWE: Hardy Weinberg Equilibrium
JNK: c-Jun N-terminal kinase
LD: Linkage disequilibrium
LVH: Left ventricular hypertrophy
MAF: Minor allele frequency
MEANS: Modular ECG Analysis System
MI: Myocardial infarction
PC: Principal component
SL: Sokolow-Lyon index
SNP: Single-nucleotide polymporphisms
SNVs: Single nucleotide variants
MAPK: Mitogen activated protein kinase family
SPEF2: Sperm flagellar protein
CNV: Copy number variations
SBP: Systolic blood pressure
DBP: Diastolic blood pressure
dbNSFP: Database of human non-synonymous SNPs and their functional predictions
