# Supplementary material for: A combined linkage, microarray and exome analysis suggests MAP3K11 as a candidate gene for left ventricular hypertrophy
Source: BMC Med Genomics. 2018 Mar 5;11:22. doi: 10.1186/s12920-018-0339-9 (PMC5838853; doi:10.1186/s12920-018-0339-9)
Supplement: Supplementary file 1 — Table S1. Coding variants under the linkage peaks for LVH proxy measurements. Table S2. Selected damaging variants in the coding regions contained in the linkage regions. Table S3. SKAT and burden tests for genes of interest. Table S4. Results of linkage analyses before (LOD1) and after (LOD2) regression on GWAS SNPs under the linkage peaks. Table S5. Descriptive statistics of the Rotterdam study population. Table S6. Replications results in the Rotterdam Study. Figure S1. Venn diagram showing the overlap between the different ERF genotyping experiments. Figure S2. Pedigrees segregating rs138968470. (DOCX 119 kb) [file 12920_2018_339_MOESM1_ESM.docx]

**A combined linkage, GWAS and exome analysis suggests *MAP3K11* as a candidate gene for left ventricular hypertrophy**

*MAP3K11* as a candidate gene for left ventricular hypertrophy

Claudia Tamar Silva^1,2,3^, Irina V. Zorkoltseva^4^, Marieke Niemeijer^5^, Marten E. van den Berg^5^, Najaf Amin^1^, Ayşe Demirkan^1^, Elisa van Leeuwen^1^, Adriana I. Iglesias^1^, Laura B Piñeros-Hernández^2^, Carlos M. Restrepo^1,2,^ Jan A. Kors^6^, Anatoly V. Kirichenko^4^, Rob Willemsen^7^, Ben A. Oostra^1,8^, Bruno H. Stricker^5,9,10^, André G. Uitterlinden^5^, Tatiana I. Axenovich^4^, Cornelia M. van Duijn^1,8^, Aaron Isaacs^1,11^

^1^Genetic Epidemiology Unit, Department of Epidemiology, Erasmus University Medical Center, Rotterdam, the Netherlands

^2^Center For Research in Genetics and Genomics (CIGGUR). GENIUROS Research Group, School of Medicine and Health Sciences. Universidad del Rosario. Bogotá, Colombia

^3^Doctoral Program in Biomedical Sciences, Universidad del Rosario, Bogotá, Colombia

^4^Institute of Cytology and Genetics SD RAS, Novosibirsk, Russia

^5^Department of Epidemiology, Erasmus University Medical Center, Rotterdam, the Netherlands

^6^Department of Medical Informatics, Erasmus University Medical Center, Rotterdam, the Netherlands

^7^Department of Clinical Genetics, Erasmus University Medical Center, Rotterdam, the Netherlands

^8^Center for Medical Systems Biology, Leiden, the Netherlands

^9^Department of Internal Medicine, Erasmus University Medical Center, Rotterdam, the Netherlands

^10^Inspectorate of Health care, The Hague, the Netherlands

^11^CARIM School for Cardiovascular Diseases, Maastricht Centre for Systems Biology (MaCSBio), and Dept. of Biochemistry, Maastricht University, Maastricht, the Netherlands

**Corresponding Author**

Aaron Isaacs, PhD

Assistant Professor

[aaron.isaacs@gmail.com](mailto:a.isaacs@erasmusmc.nl)

CARIM School for Cardiovascular Diseases

Maastricht Centre for Systems Biology (MaCSBio)

Dept. of Biochemistry

Maastricht University

Universiteitssingel 60

Room M1.14

6229ER Maastricht

the Netherlands

(T) +31 43 388 1862

**Table S1. Coding variants under the linkage peaks for LVH proxy measurements**

| **Trait** | **Locus** | **Variants in the coding region** | | | | | **Observations <= 5%** | | | **Predicted to be damaging** | | | **Genes** |
| --- | --- | --- | --- | --- | --- | --- | --- | --- | --- | --- | --- | --- | --- |
|  |  | **Synonymous** | **Missense** | **Stop** | **Splice** | **Total** | **Missense** | **Stop** | **Splice** | **Missense** | **Stop** | **Splice** |  |
| **SL** | 4q31 | 884 | 1292 | 36 | 8 | 2220 | 199 | 3 | 3 | 68 | 1 | 0 | *AFF1, ALPK1, ANK2, BANK1, CENPE, CLGN, DCHS2, EGF, ELF2, ENPEP, FAM13A, FAM190A, FAT4, FGA, FHDC1, FNIP2, FSTL5, HADH, HSD17B13, INPP4B, KIAA0922, LRBA, LRIT3, MFSD8, MGST2, PDLIM5, PET112, PLK4, PPID, PRDM5, PRMT10, PRSS12, QRFPR, RBM46, SETD7, SLC10A7, SLC39A8, SLC7A11, TBCK, TDO2, TET2, TLR2, TNIP3, USP38* |
|  | 15q25 | 388 | 435 | 5 | 2 | 830 | 57 | 1 | 1 | 19 | 0 | 0 | *CHD2, CHSY1, FANCI, HAPLN3, IGF1R, IGF1R, KIF7, LRRK1, MCTP2, MRPL46, PCSK6, PGPEP1L, PLIN1, POLG, SYNM, UNC45A* |
|  | 20p12 | 125 | 190 | 4 | 3 | 322 | 36 | 0 | 0 | 14 | 0 | 0 | *C20orf72, CSRP2BP, ENPEP, ESF1, (FLRT3, MACROD2), ISM1, NKX2-4, RALGAPA2, RIN2, SEL1L2, SLC24A3* |
| **CV** | 1p34 | 883 | 1207 | 28 | 11 | 2129 | 140 | 1 | 1 | 46 | 1 | 0 | *AK2, ATPAF1, B4GALT2, (BMP8B,PPIE), C1orf50, C1orf94, CITED4, CMPK1, COL16A1, CYP4A22, DMRTA2, EBNA1BP2, EPB41, EPHA10, FAAH, GJB4, HIVEP3, KIAA0319L, KIF2C, LEPRE1, MACF1, MAST2, MOB3C, MPL, PHACTR4, PHC2, PIK3R3, RLF, RNF220, SERINC2, SLC5A9, SPATA6, (SZT2,HYI), TINAGL1, TMCO2, TMEM125, TMEM234, UQCRH, UROD, ZYG11A* |
|  | 6q15 | 506 | 829 | 13 | 8 | 1356 | 125 | 1 | 1 | 52 | 0 | 0 | *AKD1, ANKRD6, ASCC3, BACH2, C6orf165, C6orf57, CD109, CDC40, DSE, EPHA7, FAM162B, FAM26D, FIG4, FILIP1, FRK, FYN, GPRC6A, HACE1, HTR1B, KIAA1009, LACE1, LAMA4, MDN1, ME1, MICAL1, NT5DC1, PNISR, PRDM1, RARS2, RIMS1, ROS1, RSPH4A, SCML4, SIM1, SLC17A5, SLC22A16, SNX14, SOBP, TRAF3IP2* |
| **12LS** | 5p14 | 306 | 398 | 5 | 3 | 712 | 52 | 1 | 1 | 20 | 0 | 1 | *ADAMTS12, ANKH, C5orf42, DNAH5, DROSHA, EGFLAM, FAM173B, MTRR, NADKD1, PDZD2, SEMA5A, SLC45A2, SPEF2, TARS, TRIO* |
|  | 20p12 | 582 | 377 | 23 | 9 | 991 | 127 | 1 | 1 | 40 | 0 | 0 | *ACTR5, AHCY, APMAP, ASXL1, BPIFA3, C20orf72, CHD6, CPNE1, CSRP2BP, CST8, DEFB119, DHX35, DLGAP4, E2F1, EDEM2, ENTPD6, ESF1, (FLRT3, MACROD2), ISM1, KIAA1755, LBP, LPIN3, MYH7B, MYLK2, NKX2-4, RALGAPA2, RALGAPB, RIN2, SAMHD1, SEL1L2, SLC24A3, ZNF337* |
| **PC1** | 11 | 1608 | 2526 | 51 | 35 | 4231 | 323 | 5 | 4 | 111 | 0 | 0 | *RPS6KB2, MS4A5, PRR5L, LRP4, PAMR1, OR5F1, C2CD3, SLC22A11, SLC22A24, UBXN1, LIPT2, OR10V1, CCDC88B, ZP1, PLEKHB1, NUP160, NADSYN1, PGM2L1, ZFPL1, KRTAP5-10, RSF1, RIN1, SLC22A25, GDPD4, C11orf20, OR8U1, AHNAK, RAG1, RAG2, LTBP3, UCP3, MADD, GIF, CLPB, MS4A13, RELT, OR4D6, NAT10, FERMT3, PDHX, RAPSN, OR4D9, OR9G4, OR5AP2, CLCF1, FJX1, PTPMT1, OR8H1, CTNND1, GPR137, RASGRP2, SLC22A9, TAF6L, NUMA1, ALG8, SCYL1, DGAT2, PPFIA1, INPPL1, MTL5, KRTAP5-9, PITPNM1, CD248, AGBL2, OR5A1, TMEM223, OR5J2, EXT2, TCN1, C11orf10, CAT, TMEM132A, OR4S1, EHF, CAPN1, ABTB2, CDC42BPG, DTX4, NAALADL1, SPTBN2, USP35, SLC22A10, OR5D18, MAPK8IP1, PLCB3, OR4C16, OR4C15, OR4C3, OR5M10, TSKU, DNAJC4, MAP3K11* |
|  | 20 | 475 | 724 | 21 | 9 | 1249 | 106 | 2 | 2 | 30 | 0 | 0 | *E2F1, RIN2, ASXL1, BPIFA3, MYLK2, MYH7B, NKX2-4, AHCY, CSRP2BP, CST8, ESF1, SEL1L2, EDEM2, SAMHD1, (FLRT3, MACROD2), C20orf72, CPNE1, RPN2, RALGAPA2, CSTL1, SLC24A3, ZNF337, ENTPD6, ISM1, APMAP* |
| **PC2** | 6 | 168 | 263 | 2 | 3 | 442 | 39 | 0 | 0 | 15 | 0 | 0 | *EPHA7, RARS2, C6orf165, EPHA7, BACH2, SIM1, MDN1, SNX14, PNISR, ASCC3, ANKRD6* |
|  | 9 | 143 | 276 | 5 | 2 | 435 | 48 | 0 | 0 | 12 | 0 | 0 | *PTPLAD2, DENND4C, FAM154A, PLIN2, IFNA10, FREM1, FOCAD, IFT74, IFNA14, SNAPC3* |
|  | 15 | 33 | 58 | 1 | 0 | 107 | 0 | 0 | 0 | 3 | 0 | 0 | *GOLGA6L2, CYFIP1* |
|  | 22 | 844 | 1107 | 14 | 13 | 2000 | 174 | 1 | 4 | 41 | 0 | 0 | *PLA2G3, FOXRED2, SMTN, GRAP2, MFNG, C1QTNF6, SAMM50, PACSIN2, GCAT, MYO18B, EMID1, PRR14L, SMCR7L, ZC3H7B, DEPDC5, SUN2, CRYBA4, BPIFC, SEZ6L, GALR3, SFI1, TMEM184B, SEC14L4, XRCC6, SLC5A4, ELFN2, PNPLA5, TMPRSS6, ASCC2, CSF2RB, TCF20, (XPNPEP3, DNAJB7), MCM5, LIMK2, GAL3ST1* |

SL: Sokolow-Lyon; CV: Cornell Voltage product; 12LS: twelve-lead sum product. PC1: first principal component; PC2: second principal component.

**Table S2**. **Selected damaging variants in the coding regions contained in the linkage regions**

| **Trait** | **chr:pos** | **Alleles** | **Freq** | **Alleles** | **n** | **Beta** | **SE** | ***P*** | ***P*_HWE_** | **rsID** | **Gene** |
| --- | --- | --- | --- | --- | --- | --- | --- | --- | --- | --- | --- |
| CV | 1:32149760 | C/**T** | 2.02E-02 | 43 | 1066 | 0.27 | 0.14 | 4.96E-02 | 0.01 | rs34770879 | *COL16A1* |
| CV | 1:33487277 | **C**/T | 3.50E-02 | 75 | 1070 | 0.25 | 0.12 | 3.31E-02 | 0.14 | rs184683619 | *AK2* |
| CV | 1:40717144 | C/**G** | 1.26E-02 | 27 | 1070 | -0.41 | 0.19 | 2.78E-02 | 1.00 | rs80094747 | *TMCO2* |
| CV | 1:45110913 | **A**/G | 1.17E-02 | 25 | 1070 | -0.51 | 0.20 | 1.03E-02 | 1.00 | - | *RNF220* |
| CV | 1:46487706 | C/**G** | 1.17E-02 | 25 | 1070 | 0.46 | 0.20 | 2.44E-02 | 1.00 | rs201214757 | *MAST2* |
| SL | 4:91234118 | C/**T** | 1.12E-02 | 24 | 1070 | -0.47 | 0.20 | 1.99E-02 | 1.00 | rs142352144 | *FAM190A* |
| SL | 4:119203330 | **A**/G | 2.99E-02 | 64 | 1070 | 0.26 | 0.13 | 4.22E-02 | 0.62 | rs35996030 | *PRSS12* |
| SL | 4:119216099 | **C**/G | 1.54E-02 | 33 | 1070 | -0.60 | 0.18 | 8.40E-04 | 1.00 | rs142551296 | *PRSS12* |
| SL | 4:151520216 | **A**/G | 2.20E-02 | 47 | 1070 | -0.34 | 0.14 | 1.56E-02 | 0.40 | rs35879351 | *LRBA* |
| 12LS | 5:13914743 | A/**T** | 1.12E-02 | 24 | 1070 | -0.50 | 0.20 | 1.18E-02 | 1.00 | rs140782270 | *DNAH5* |
| 12LS | 5:356672 | **A**/G | 5.05E-02 | 108 | 1070 | -0.34 | 0.10 | 4.14E-04 | 0.34 | rs139580877 | *SPEF2* |
| PC2 | 6:94120219 | **A**/G | 1.22E-02 | 24 | 981 | -0.43 | 0.20 | 3.22E-02 | 1.00 | rs2278106 | *EPHA7* |
| PC2 | 9:15459821 | **A/C** | 4.99E-02 | 98 | 981 | 0.21 | 0.10 | 3.72E-02 | 1.00 | rs3087653 | *SNAPC3* |
| PC1 | 11:48347082 | **A/G** | 1.02E-02 | 20 | 981 | -0.48 | 0.22 | 2.90E-02 | 1.00 | rs148047717 | *OR4C3* |
| PC1 | 11:55322606 | **C**/T | 4.23E-02 | 83 | 981 | -0.23 | 0.11 | 3.28E-02 | 0.03 | rs17581700 | *OR4C15* |
| PC1 | 11:55339704 | **G**/T | 4.23E-02 | 83 | 981 | -0.23 | 0.11 | 3.28E-02 | 0.03 | rs78612916 | *OR4C16* |
| PC1 | 11:56344846 | **A**/T | 3.16E-02 | 62 | 980 | -0.28 | 0.12 | 2.00E-02 | 0.01 | - | *OR5M10* |
| PC1 | 11:62286666 | **C/T** | 1.43E-02 | 28 | 981 | 0.42 | 0.19 | 2.47E-02 | 1.00 | rs114515655 | *AHNAK* |
| PC1 | 11:63999929 | **C/T** | 1.43E-02 | 28 | 981 | -0.49 | 0.20 | 1.37E-02 | 1.00 | rs199745306 | *DNAJC4* |
| PC1 | 11:64031030 | **A/**G | 1.22E-02 | 24 | 981 | 0.42 | 0.21 | 3.99E-02 | 1.00 | rs145502455 | *PLCB3* |
| PC1 | 11:65373252 | **A/G** | 1.22E-02 | 24 | 981 | 0.76 | 0.20 | 2.03E-04 | 0.13 | rs138968470 | *MAP3K11* |
| PC1 | 11:76507144 | **G/T** | 3.47E-02 | 68 | 981 | -0.31 | 0.13 | 1.80E-02 | 0.63 | rs148661218 | *TSKU* |
| SL | 15:91496242 | C/**T** | 1.31E-02 | 28 | 1068 | -0.44 | 0.18 | 1.60E-02 | 1.00 | rs8041035 | *UNC45A* |
| PC1 | 20:14306773 | **G**/T | 4.55E-02 | 89 | 978 | -0.24 | 0.11 | 3.00E-02 | 1.00 | rs35253731 | *FLRT3, MACROD2* |
| SL | 20:14306773 | **G**/T | 4.69E-02 | 100 | 1067 | -0.23 | 0.10 | 2.34E-02 | 1.00 | rs35253731 | *FLRT3, MACROD2* |
| SL | 20:18162405 | **A**/T | 1.08E-02 | 23 | 1067 | -0.43 | 0.21 | 4.21E-02 | 1.00 | rs139667492 | *CSRP2BP* |
| 12LS | 20:36993333 | A/**G** | 2.16E-02 | 46 | 1067 | -0.29 | 0.14 | 3.96E-02 | 0.08 | rs2232607 | *LBP* |
| PC2 | 22:30951503 | **C/T** | 1.64E-02 | 32 | 978 | 0.42 | 0.17 | 1.47E-02 | 0.23 | rs151043823 | *GAL3ST1* |
| PC2 | 22:31671227 | **C/T** | 1.99E-02 | 39 | 978 | -0.39 | 0.16 | 1.60E-02 | 0.32 | - | *LIMK2* |

Effect allele in bold. chr:pos: chromosome:position; n: sample size; Beta: effect estimate; *P: P*-value; *P*_HWE_: Hardy-Weinberg *P*-value; SL: Sokolow-Lyon; CV: Cornell Voltage product; 12LS: twelve-lead sum product; PC1: first principal component; PC2: second principal component.

**Table S3. SKAT and burden tests for genes of interest**

|  | **CV** | |  | **SL** | |  | **12LS** | |
| --- | --- | --- | --- | --- | --- | --- | --- | --- |
| **Gene** | ***P*_SKAT_** | ***P*_Burden_** |  | ***P*_SKAT_** | ***P*_Burden_** |  | ***P*_SKAT_** | ***P*_Burden_** |
| *MAP3K11* | 8.07 x 10^-2^ | 4.19 x 10^-1^ |  | 5.50 x 10^-2^ | 6.45 x 10^-1^ |  | 2.82 x 10^-2^ | 8.76 x 10^-1^ |
| *PRSS12* | 4.95 x 10^-1^ | 7.68 x 10^-2^ |  | 3.02 x 10^-2^ | 7.91 x 10^-3^ |  | 7.24 x 10^-2^ | 4.97 x 10^-2^ |
| *SPEF2* | 2.72 x 10^-1^ | 8.70 x 10^-1^ |  | 5.10 x 10^-1^ | 7.83 x 10^-1^ |  | 6.36 x 10^-1^ | 4.09 x 10^-1^ |

SL: Sokolow-Lyon; CV: Cornell Voltage product; 12LS: twelve-lead sum product; *P*_SKAT_: *P*-value for SKAT –o; *P*_Burden_: *P*-value for burden test. Burden tests performed for variants with minor allele frequency < 0.25.

**Table S4. Results of linkage analyses before (LOD1) and after (LOD2) regression on GWAS SNPs under the linkage peaks**

| **Trait** | **Chr** | **Position (cM)** | **LOD1 (n)** | **LOD2 (n)** | **SNP** |
| --- | --- | --- | --- | --- | --- |
| CV | 1 | 59.63 | 2.4 (1853) | 2.09 (1783) | rs17391905 |
| SL | 15 | 112.30 | 1.92 (1854) | 1.62 (1783) | rs8038015 |
| 12LS | 5 | 42.30 | 2.18 (1855) | 2.24 (1783) | rs13185595 |
| 12LS | 20 | 40.70 | 2.12 (1855) | 2.31 (1783) | rs2025096 |
| PC1 | 11 | 65.21 | 2.01 (1394) | 1.49 (1340) | rs2269434 |
| PC1 | 11 | 65.21 | 2.01 (1394) | 1.60 (1340) | rs174577 |

Chr: chromosome; cM: centimorgan; n: sample size; LOD1: initial LOD score; LOD2: lod score conditioned on SNP; SNP: GWAS index SNPs under the linkage peaks; SL: Sokolow-Lyon; CV: Cornell Voltage product; 12LS: twelve-lead sum product; PC1: first principal component.

**Table S5. Descriptive statistics of the Rotterdam study population**

|  | **Exome Sequence** | | | **RS** | | |
| --- | --- | --- | --- | --- | --- | --- |
|  | **n = 1450** | | | **n = 5783** | | |
|  | **Mean (S.D.)** | **Minimum** | **Maximum** | **Mean (S.D.)** | **Minimum** | **Maximum** |
| Males | 648 (44.7%) |  |  | 2364 (40.9%) |  |  |
| Age (y) | 68.6 (8.52) | 55.1 | 101.8 | 69.0 (8.72) | 55.1 | 107.3 |
| BMI (kg/m^2^) | 26.5 (3.58) | 15.4 | 42.5 | 26.3 (3.67) | 14.3 | 50.7 |
| Height (cm) | 167.5 (9.44) | 137 | 198 | 167.0 (9.34) | 137 | 198 |
| Weight (kg) | 74.3 (11.7) | 40.1 | 115.1 | 73.4 (11.88) | 40.0 | 146.5 |
| SBP (mm Hg) | 139.7 (22.29) | 84 | 245 | 139.1 (22.3) | 79 | 250 |
| DBP (mm Hg) | 74.17 (11.45) | 36 | 139 | 73.7 (11.4) | 36 | 139 |
| Hypertension | 792 (54.6%) |  |  | 3150 (54.5%) |  |  |
| SL | 2326.0 (758.5) | 223 | 6174 | 2341.0 (751.5) | 223 | 7052 |
| CV | 1481.1 (593.4) | 36 | 5273 | 1471.27 (603.2) | 36 | 5273 |
| 12LS | 14511 (3323.8) | 6859 | 31997 | 14526 (3301.4) | 6201 | 31997 |
| LVH (SL) | 114 (7.9%) |  |  | 433 (7.5%) |  |  |
| LVH (CV) | 266 (18.3%) |  |  | 1155 (20%) |  |  |
| LVH (12LS) | 145 (10.0%) |  |  | 566 (9.8%) |  |  |

RS: Rotterdam Study; n: sample size; S.D.: standard deviation; BMI: Body Mass Index, SBP: Systolic blood pressure, DBP: Diastolic blood pressure, SL: Sokolow-Lyon index, CV: Cornell product, 12LS: 12-lead sum product.

Values presented are mean (standard deviation) or n (%).

**Table S6. Replications results in the Rotterdam Study**

|  |  | **RS** |  |  |  |
| --- | --- | --- | --- | --- | --- |
| SNP | Trait | MAF | β | SE | *P* |
| rs139580877* | CV | 7.90x10^-3^ | 0.00 | 0.00 | 0.15 |
|  | SL |  | 0.00 | 0.00 | 0.24 |
|  | 12LS |  | 0.00 | 0.00 | 0.21 |
| rs138968470 | CV | 2.4x10^-1^ | 0.00 | 0.00 | 0.82 |
|  | SL |  | 0.00 | 0.00 | 0.82 |
|  | 12LS |  | 0.00 | 0.00 | 0.30 |
| rs142551296 | CV | 3.6x10^-2^ | -0.09 | 0.06 | 0.10 |
|  | SL |  | -0.05 | 0.06 | 0.35 |
|  | 12LS |  | -0.06 | 0.06 | 0.31 |
| rs35996030 | CV | 0.5x10^-3^ | -0.08 | 0.06 | 0.16 |
|  | SL |  | -0.04 | 0.06 | 0.48 |
|  | 12LS |  | -0.05 | 0.06 | 0.43 |

RS: Rotterdam Study; MAF: Minor Allele Frequency; β: Effect Estimate; SE: Standard Error; *P*: *P*-value; CV: Cornell Voltage Product; SL: Sokolow-Lyon; 12LS: 12-lead Sum Product; *: imputed to 1000 Genomes.

**Figure S1. Venn diagram showing the overlap between the different ERF genotyping experiments**


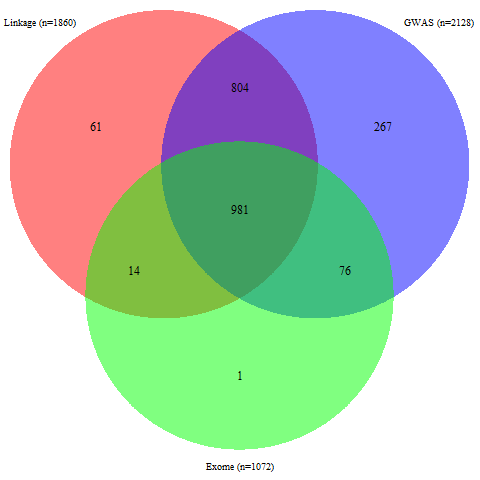


Total sample sizes for each of the three genotyping experiments are given in the labels for each circle. Overlaps between the three datasets are provided in the corresponding portion of the diagram.

**Figure S2. Pedigrees segregating rs138968470**


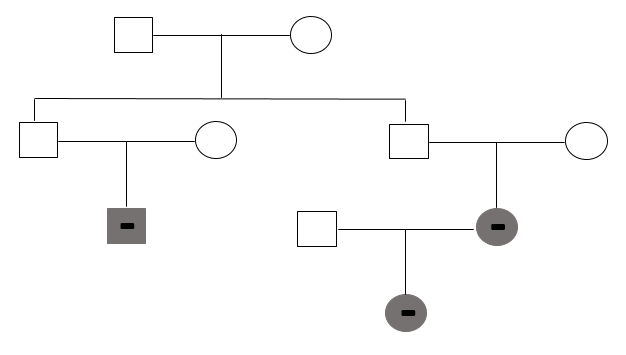


**Pedigree2**


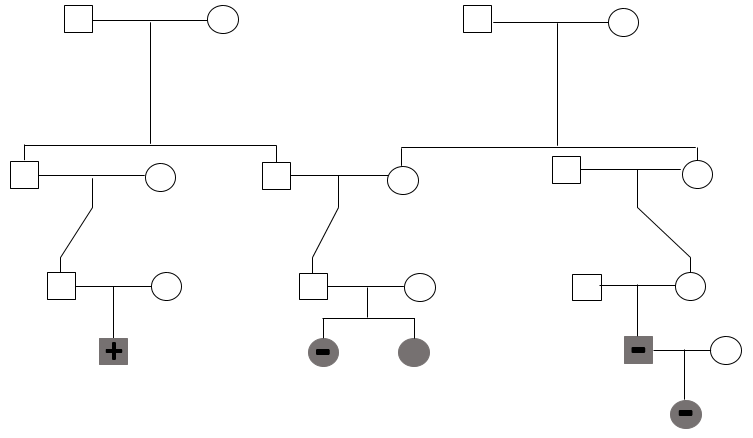


**Pedigree3**


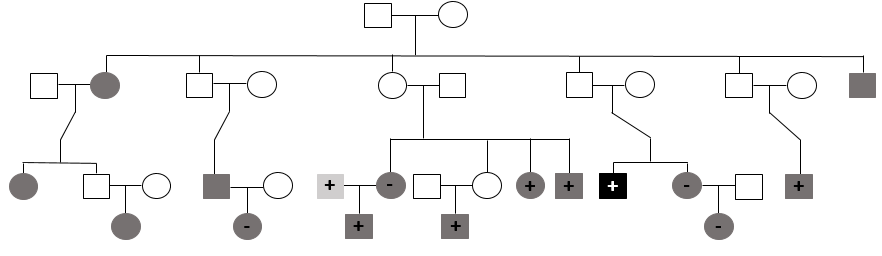


**Pedigree4**


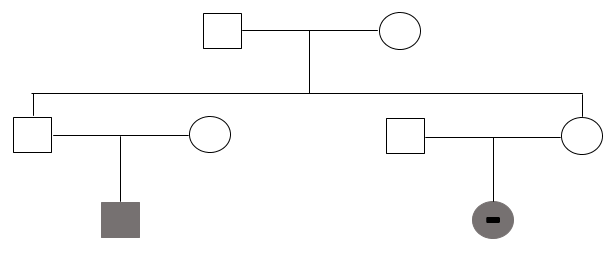


**Pedigree5**


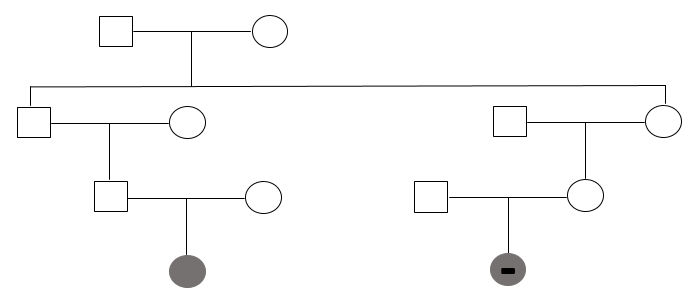


Five pedigrees segregating rs138968470 are depicted. Square: males; circle: females; clear symbols: not genotyped; light gray symbols: non-carrier; dark gray symbols: heterozygous carriers; black symbols: homozygous carriers; +: LVH cases (by proxy measure cut-off); ˗: non-LVH cases (by proxy measure cut-off).
